# Therapeutic Effects of *Pimpinella anisum* Fruit Extract on Polycystic Ovary Syndrome in a Rat Model: Emerging Role of Inflammatory Responses and Oxidative Stress

**DOI:** 10.5812/ijpr-143290

**Published:** 2024-03-09

**Authors:** Masoomeh Dadkhah, Negin Gholizadeh, Ramin Nasimi Doost Azgomi, Shahnaz Hosseinzadeh, Sanaz Hamedeyazdan, Khadijeh Haghighat, Salva Afshari, Mina Salimi, Arezoo Moini Jazani

**Affiliations:** 1 Pharmaceutical Sciences Research Center, Ardabil University of Medical Sciences, Ardabil, Iran; 2 Student Research Committee, Health School, Ardabil University of Medical Sciences, Ardabil, Iran; 3 Traditional Medicine and Hydrotherapy Research Center, Ardabil University of Medical Sciences, Ardabil, Iran; 4 Department of Microbiology & Immunology, Faculty of Medicine, Ardabil University of Medical Sciences, Ardabil, Iran; 5 Department of Pharmacognosy, Faculty of Pharmacy, Tabriz University of Medical Sciences, Tabriz, Iran; 6 Department of Biology, Faculty of Sciences, University of Mohaghegh Ardabili, Ardabil, Iran; 7 Students Research Committee, Pharmacy School, Ardabil University of Medical Sciences, Ardabil, Iran

**Keywords:** *Pimpinella anisum*, Polycystic Ovary Syndrome, Oxidative Stress, Inflammation, Rat

## Abstract

**Background:**

Polycystic ovary syndrome (PCOS) is the most common gynecological endocrine disorder.

**Objectives:**

This study evaluated the therapeutic effects of *Pimpinella anisum* L. (*P. anisum*) fruit on pro-inflammatory cytokines, oxidative stress markers, and ovarian tissue structure in a rat model of PCOS.

**Methods:**

After inducing PCOS, female Wistar rats were randomly divided into control and PCOS groups. They orally received daily doses of normal saline or hydro-alcoholic extract of *P. anisum* at two doses (200 and 400 mg/kg) for 21 days. At the end of the treatment period, ovarian and liver tissues were collected to measure lipid peroxidation, antioxidant status, TNF-α, IL-6 mRNA expression, and its content. Additionally, histopathological examinations of the ovarian tissue were conducted.

**Results:**

Our findings revealed a dose-dependent change in the biochemical and histopathological parameters. Treatment with *P. anisum* resulted in a significant decrease in TNF-α and IL-6 mRNA expression levels and their content in the ovarian and liver tissues. It also reduced MDA levels while increasing SOD and GPx activity in both ovarian and liver tissues of PCOS rats. Furthermore, the number of follicular cysts in the PCOS rat model was significantly reduced.

**Conclusions:**

The beneficial effects of *P. anisum* in PCOS rats are partly attributed to the inhibition of inflammatory and oxidative stress markers in ovarian tissue. These findings suggest that *P. anisum* could be a potential candidate for the treatment of PCOS disorders

## 1. Background

Polycystic ovary syndrome (PCOS) is a complex endocrine disorder in women of fertile age, affecting approximately 5 to 10% of women worldwide (1, 2). It is clinically characterized by menstrual irregularity, hyperandrogenism (such as hirsutism and acne), obesity, and chronic oligo- or anovulation (3, 4). Studies suggest that about 15% of women experiencing PCOS during their reproductive years are at high risk of various health problems, including metabolic, reproductive, and endocrine disorders (5, 6). Despite extensive research, the pathophysiology of PCOS remains incompletely understood. PCOS is a multifactorial disorder involving abdominal obesity (7), hormonal disturbances (8), insulin resistance (9), and the interplay of epigenetic and environmental factors (10).

Oxidative stress, defined as a disturbance in the balance between oxidants and antioxidants resulting in excessive free radical (ROS) formation at the cellular level, contributes to ovarian follicle development disruption, impairment of ovarian tissue, DNA damage, and cell apoptosis (11, 12). Elevated serum lipid peroxide levels, inflammatory markers (13-15), and macrophage infiltration in peripheral tissues are commonly observed in patients with PCOS, leading to tissue damage and impaired function. These parameters are considered potential triggers of reproductive diseases, including PCOS (16, 17). Understanding the interconnected effects of inflammation and oxidative capacity associated with PCOS is crucial for identifying and managing this ovarian disease.

Over the past years, numerous studies have explored the potential benefits of polyphenols in addressing reproductive disorders (18, 19). *Pimpinella anisum* L. (*P. anisum*), commonly known as aniseed or anise, is an aromatic plant belonging to the Umbelliferae (Apiaceae) family, primarily found in the Middle East, Asia Minor, and India (20, 21). Aniseed extract contains various compounds, including 1.5 - 6% essential oil (mainly trans-anethole), 8 - 11% fatty acids (such as palmitic and oleic acids), 4% carbohydrates, and 18% protein, along with significant amounts of phenolic compounds, such as flavonoids and phenolic acids (22). Several studies have highlighted the pharmacological properties of *P. anisum* fruits, including anti-inflammatory, antioxidant, hepatoprotective, dysmenorrhea-relieving, hypolipidemic, and anti-diabetic activities.

Recent research has indicated that *P. anisum* exhibits estrogenic effects, promoting the development of ovarian follicles, restoring ovarian anatomy, reducing menopausal hot flashes, and altering levels of luteinizing hormone (LH) (23-26).

## 2. Objectives

Although earlier studies have reported the efficacy of *P. anisum* in PCOS, they have primarily focused on its effects on hormonal and biochemical factors associated with PCOS. Given the lack of research on the effects of aniseed extract on inflammatory and oxidative stress factors in PCOS tissue, the current study was designed to investigate the impact of *P. anisum* fruit hydroalcoholic extract on inflammatory and oxidative stress markers, as well as ovarian tissue structure, in a rat PCOS model.

## 3. Methods

### 3.1. Animals

In the current experiment, twenty female Wistar rats (8 - 10 weeks old), weighing 200 ± 20 g, were obtained from the central animal house of the Pasteur Institute of Iran (Tehran, Iran). The animals were group-housed (2 rats/cage) in a temperature-controlled room at 22 ± 2°C under a 12:12 h light/dark cycle, with at least 1 week of local adaptation prior to experimental use. Standard pellets (Pars Company, Tehran, Iran) and water were provided ad libitum. All experimental procedures were conducted in accordance with NIH guidelines and approved by the Ethics Committee of Ardabil University of Medical Sciences (Ethics code: IR.ARUMS.REC.1400.183).

### 3.2. Plant Material Preparation, Extraction, and Qualitative Screening

Fruits of *P. anisum* were purchased from the local herbal market in Tabriz. After identification and confirmation of the plant materials by the herbarium of the Department of Pharmacognosy, Faculty of Pharmacy, Tabriz University of Medical Sciences, with voucher herbarium specimen (No.tbz-fps-45), they were dried and powdered using a blender. Subsequently, the powdered plant materials were macerated with 3 L of 70% ethanol in triplicate for a cycle of 48 h. Finally, the resulting extract was filtered, and the solvent was evaporated using a rotary evaporator under vacuum at 40˚C until completely dried. Several qualitative and quantitative phytochemical assays on the *P. anisum* extract were performed following standard practices as designated by Sofowora (27) and Trease and Evans (28).

### 3.3. Assay for Free Radical Scavenging Activity

The free radical scavenging activity of *P. anisum* fruit extract was evaluated using 2,2-diphenyl-1-picrylhydrazyl (DPPH). Initially, a stock solution (1 mg/mL) of *P. anisum* extract was prepared, and then different solutions of the extract were prepared using a serial dilution method. Equal volumes of the prepared concentrations of the *P. anisum* extract were added to a DPPH solution with a concentration of 0.08% (29, 30). After incubating for 30 minutes, the absorbance of all solutions was measured at 517 nm alongside a blank sample. Finally, the percentage of DPPH radical scavenging activity of the *P. anisum* extract was calculated using the following formula:


$$R\;\left(\%\right)=\;100\times [\frac{\left(Absorbance\;blank-Absorbance\;sample\right)}{Absorbance\;blank}]$$

Furthermore, the concentration of the extract required to reduce 50% of the DPPH free radicals, representing RC50, was determined from the graph of reduction percentages (R%) versus concentrations of *P. anisum* extract.

### 3.4. Assays for Total Phenol and Flavonoid Contents

Using the Folin-Ciocalteu assay, the total phenol content was measured in 100 g of dried *P. anisum* fruit material. Initially, 500 µL of the stock solution (1 mg/mL) of *P. anisum* extract was mixed with 5 mL of Folin-Ciocalteu reagent (10%, v/v) and 4 mL of 1 M sodium bicarbonate (31). After incubating for 15 minutes at 25ºC, the UV absorbance of the formed blue-colored solution was read at 765 nm. Gallic acid was used as the standard phenol compound, and the same process was repeated for its solutions. The total phenolic content in the *P. anisum* fruit extract was calculated based on the depicted gallic acid calibration curve.

In an investigation to confirm the total flavonoid content in 100 g of dried *P. anisum* fruit material, 500 µL of the stock solution (1 mg/mL) of *P. anisum* extract was mixed with 1.5 mL methanol, 2.8 mL distilled water, 0.1 mL aluminum chloride (10%), and 0.1 mL potassium acetate. After incubating for 30 minutes, the UV absorbance of the formed yellow-colored solution was read at 435 nm (29). Quercetin was used as the standard phenol compound, and the same process was repeated for its solutions. The total flavonoid content in the *P. anisum* fruit extract was calculated based on the depicted quercetin calibration curve.

### 3.5. Induction of PCOS Model

The PCOS rat model was induced experimentally by a single intramuscular injection of estradiol valerate (EV) during the estrous stage based on Brawer et al. (32) with slight modifications (EV, 2 mg/kg dissolved in 0.2 mL sesame oil, Aburaihan Pharmaceutical, Iran). Sixty days after the EV injection, vaginal smear tests were conducted to determine the abnormality of the estrous cycles and the presence of persistent vaginal cornification epithelial cells (PVC), which is one of the symptoms of ovarian cysts.

### 3.6. Experimental Design

**Figure 1.**
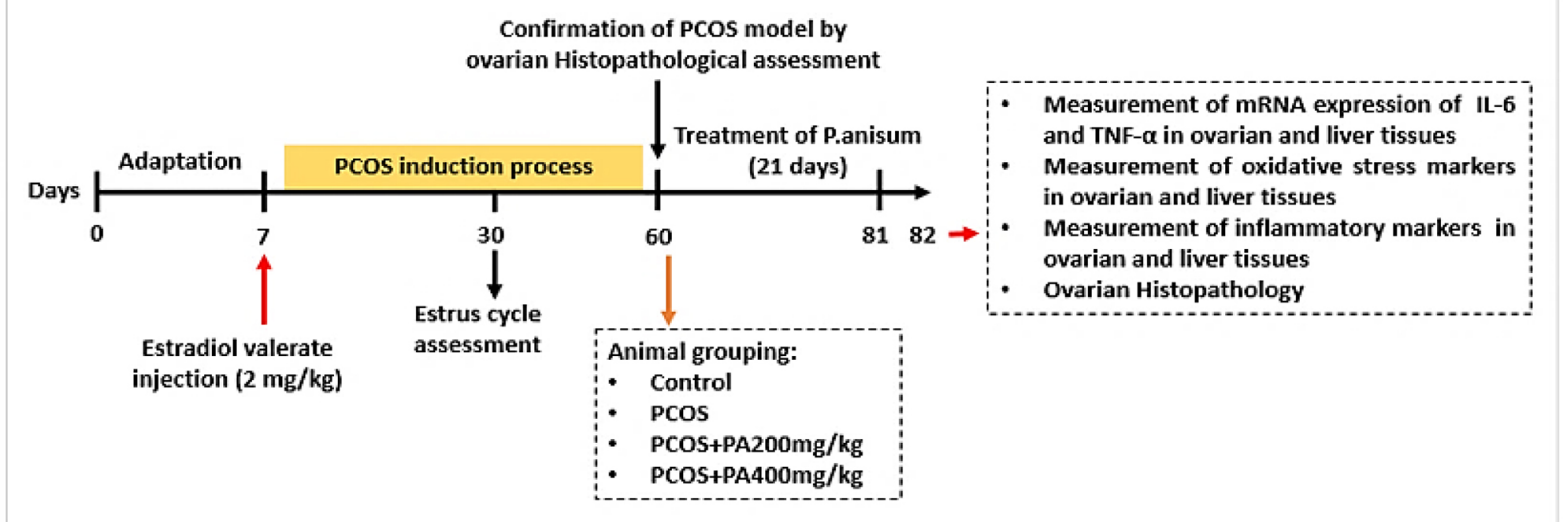
Experimental design

Female Wistar rats were randomly assigned to four groups (n = 5/group), including: (1) Control group: Non-PCOS induced rats that received tap water and food; (2) PCOS group: PCOS rats that received 0.5 mL/day normal saline orally (gavage) for 3 weeks; (3) Polycystic ovary syndrome + *P. anisum* 200 mg/kg group (PCOS + PA200): PCOS rats received *P. anisum* extract at a dose of 200 mg/kg orally once daily for 3 weeks; (IV) PCOS + *P. anisum* 400 mg/kg group (PCOS + PA400): PCOS rats received *P. anisum* extract at a dose of 400 mg/kg orally once daily for 3 weeks (26). Figure 1 illustrates the experimental design.

### 3.7. Tissue Sampling

After 21 days of *P. anisum* administration, all animals were anesthetized with an intraperitoneal (IP) injection of ketamine/xylazine (80/10 mg/kg) and sacrificed by cervical dislocation. The liver and ovaries of the rats were isolated and weighed. The right ovary and liver were homogenized in lysis buffer, centrifuged at 1250 × g for 30 min at 4°C (33), and the supernatants were collected and kept at −80°C until analysis. The left ovaries were fixed in a 10% formalin solution for histopathological examination.

### 3.8. Measurement of the TNF-α and IL-6 Levels in Ovarian and Liver Tissues

The levels of tumor necrosis factor-α (TNF-α) and interleukin 6 (IL-6) in tissue homogenates were measured using a rat ELISA kit (DuoSet, R&D Systems, China). Assays were performed according to the manufacturer’s instructions.

### 3.9. Measurement of the MDA Levels and SOD and GPx Activities in Ovarian and Liver Tissues

The malondialdehyde (MDA) content in the supernatant of the homogenate was determined using a commercial colorimetric kit (ZellBio GmbH, Ulm, Germany) following the manufacturer’s protocols. To measure superoxide dismutase (SOD) activity in tissue homogenate supernatant, the Ransod kit (Randox Labs, Crumlin, UK) was used according to the manufacturer’s instructions, and the activity was expressed as U/mg protein. Glutathione peroxidase (GPx) activity in tissue homogenates was assayed with the GPx assay kit (ZellBio GmbH, Ulm, Germany), and the results were expressed as U/mg.

### 3.10. Quantitative Real-Time PCR (qRT-PCR)

The relative expression of TNF-α and IL-6 genes was measured in ovarian and liver tissues, with the GAPDH gene serving as the reference gene. Total RNA was extracted from tissue samples using Trizol One Step RNA Reagent (Cat. No. BS410A, Bio Basic, Canada). A NanoDrop spectrophotometer (Thermo Scientific TM NanoDrop 2000, USA) was used to assess the quality of the isolated RNA. The synthesis of cDNA was performed using the cDNA synthesis kit (YTA, Iran) and the T100TM Bio-Rad 96-Well Thermal Cycler. Real-time PCR was performed utilizing the SYBR Green Real-Time PCR Master Mix (YTA, Iran) and the Real-Time PCR Step One Plus (Applied Biosystems, Thermo Fisher Scientific, USA). Primer sequences are listed in Table 1. The Real-Time PCR procedure involved pre-incubation at 95°C for 10 minutes, followed by 40 cycles of 95°C for 15 seconds, 60°C for 30 seconds, and 72°C for 25 seconds. The qRT-PCR results were analyzed by the 2^-ΔΔCt ^method.

**Table 1.** List of the Primer Sequences Used for qRT-PCR

Genes	Primer Sequence
**TNF-α**	
Forward	5́ ATCGGTCCCAACAAGGAGGA 3́
Reverse	5́ TCCGCTTGGTGGTTTGCTAC 3́
**IL-6**	
Forward	5́ GACTTCCAGCCAGTTGCCTTCTTG3́
Reverse	5́ TGGTCTGTTGTGGGTGGTATCCTC 3́
**GAPDH**	
Forward	5́ CCTCCAGGAGCGAGATCC C 3́
Reverse	5́ GTGGTTCACACCCATCACAAA C 3́

### 3.11. Ovarian Histopathology

Ovarian samples were removed and placed in 10% formalin for 24 hours at 4°C, dehydrated in a series of graded ethanol, and embedded in paraffin blocks. Then, 5-µm-thick longitudinal sections were prepared by a microtome and, finally, stained with hematoxylin and eosin (Merck, Germany) to investigate the number of cysts in the ovaries of each group per section using a light microscope equipped with a digital camera (34).

### 3.12. Statistical Analysis

All data were analyzed using GraphPad Prism software, version 8.0, and expressed as mean ± standard error of the mean (SEM). Results obtained from the analysis of inflammatory and oxidative stress markers, histopathological examination, as well as molecular results were subjected to one-way analysis of variance (ANOVA), followed by Tukey’s post-hoc test to assess comparisons between experimental groups. A P-value < 0.05 was considered statistically significant.

## 4. Results

### 4.1. Phytochemical Analysis

Qualitative tests revealed the presence of several groups of compounds, including alkaloids, flavonoids, saponins, and tannins, in the methanol extract of *P. anisum*. Additionally, cardiac glycosides and coumarins were found to be absent in the extract of *P. anisum* fruits. The results of the in vitro free radical scavenging assay demonstrated that the extract of *P. anisum* fruits exhibited satisfactory antioxidant activity compared to the standard quercetin. The RC50 value for the DPPH antioxidant activity of the extract was determined as 304.5 µg/mL compared to the standard quercetin with a value of 3.7 µg/mL as the positive control. Furthermore, the total phenolics and flavonoid contents were calculated as 5.1 g gallic acid equivalent and 3.4 g quercetin equivalent per 100 g of dried *P. anisum* fruits. Therefore, the obtained results for DPPH radical scavenging activity were consistent with our data for total phenolics and flavonoids content, suggesting that the high contents of phenolic compounds in *P. anisum* fruits partly contributed to the observed in vitro antioxidant activity.

### 4.2. Effects of *P. anisum* Extract on Inflammatory Cytokines in Ovarian Tissue of PCOS-Induced Rats

**Figure 2.**
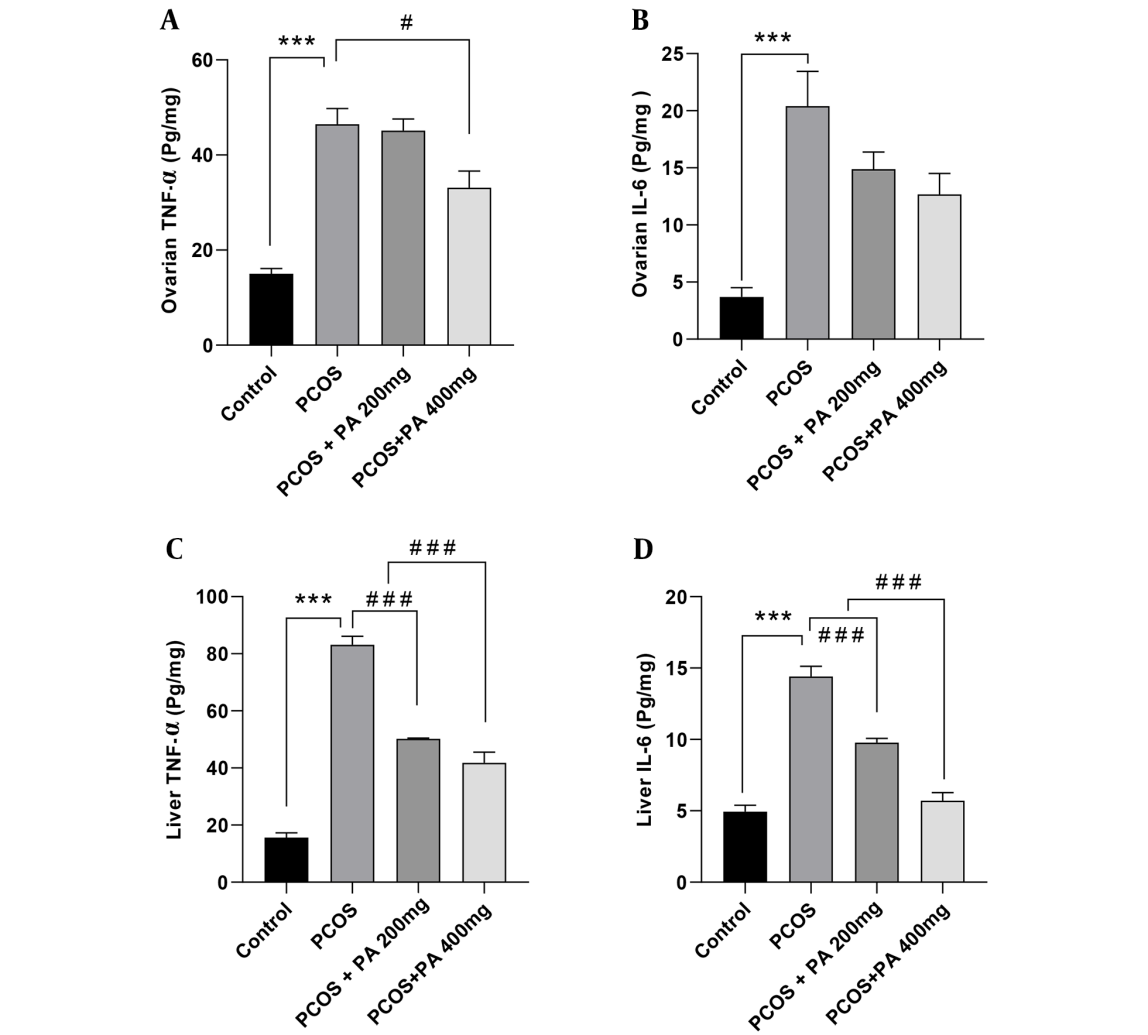
Effect of treatment with *P. anisum* extract (200 and 400 mg/kg, orally, once daily, for 3 weeks) on TNF-α and IL-6 levels in ovarian (A and B) and liver (C and D) tissues of PCOS-induced rats. Values are expressed as mean ± SEM (n = 5 per group). Data were analyzed using one-way ANOVA followed by Tukey’s post hoc test. *** P < 0.001 vs. the control group and # P < 0.05, ### P < 0.001 vs. the PCOS group. PCOS: Polycystic ovary syndrome group; PA: *Pimpinella anisum*.

As shown in Figure 2A, ovarian TNF-α levels in the PCOS group were significantly elevated compared to controls (P < 0.001). Additionally, in the group treated with a 400 mg/kg dose of *P. anisum*, ovarian TNF-α levels were markedly decreased compared to the PCOS group (Figure 2A, P < 0.05). Ovarian IL-6 levels in the PCOS group were significantly higher compared with the control group (Figure 2B, P < 0.001). Furthermore, in the groups receiving 200 and 400 mg/kg *P. anisum*, IL-6 levels showed a non-significant decrease compared with the PCOS group.

### 4.3. Effects of *P. anisum* Extract on Inflammatory Cytokines in Liver Tissue of PCOS-Induced Rats

According to Figure 2C and D, the levels of TNF-α and IL-6 in the liver were significantly higher in the PCOS group compared to the control group (P < 0.001). Moreover, treatment with both 200 and 400 mg/kg doses of *P. anisum* resulted in a significant decrease in the liver levels of TNF-α and IL-6 compared to the PCOS group (Figure 2C and D, P < 0.001).

### 4.4. Effects of *P. anisum* Extract on Oxidative Stress Biomarkers in Ovarian Tissue of PCOS-Induced Rats

**Figure 3.**
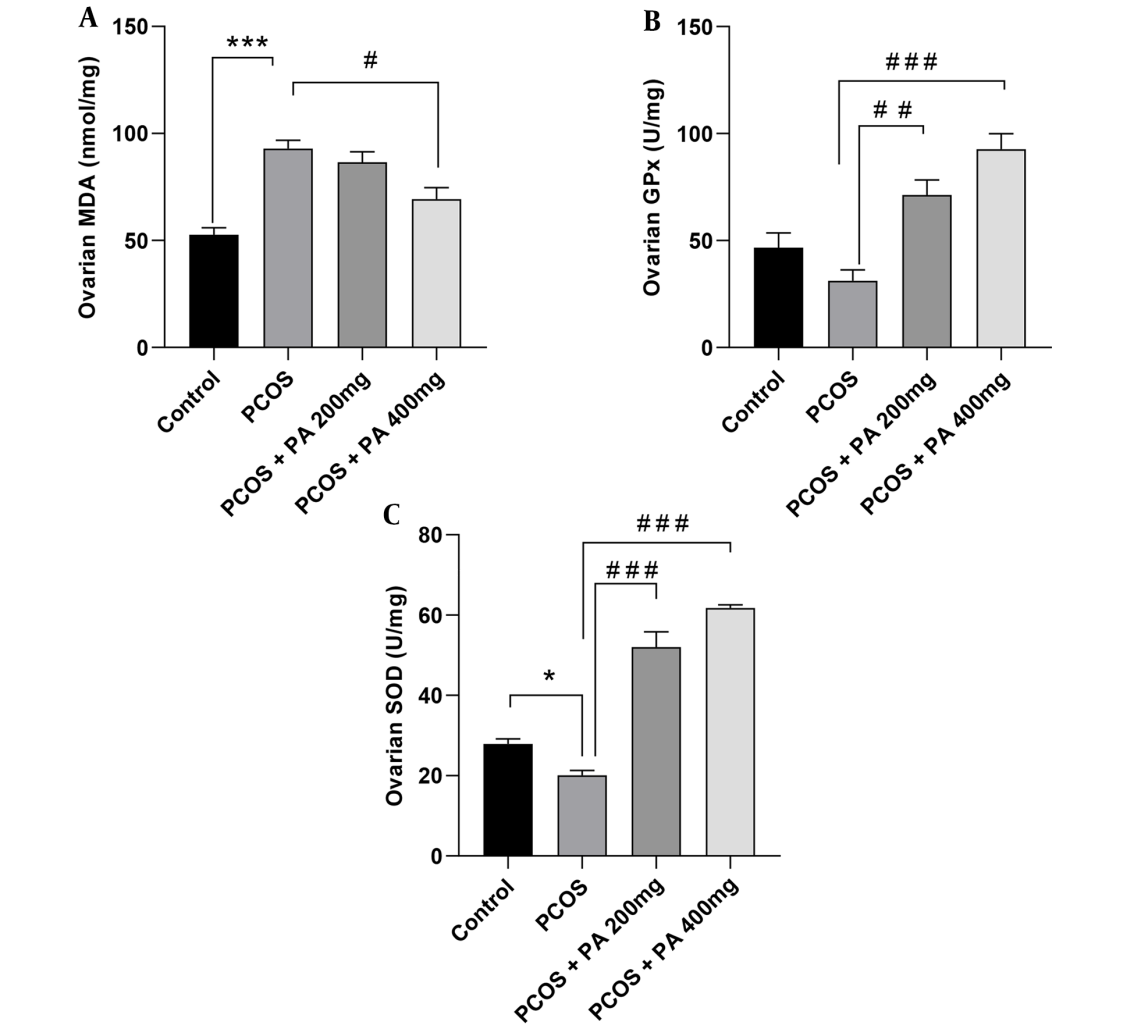
Effect of treatment with *P. anisum* extract (200 and 400 mg/kg, orally, once daily, for 3 weeks) on MDA (A) levels, GPx (B), and SOD (C) activities in ovarian tissue of PCOS-induced rats. Values are expressed as mean ± SEM (n = 5 per group). Data were analyzed using one-way ANOVA followed by Tukey’s post hoc test. * P < 0.05, *** P < 0.001 vs. the control group and # P < 0.05, ## P < 0.01, ### P < 0.001 vs. the PCOS group. PCOS: Polycystic ovary syndrome group; PA: *Pimpinella anisum*.

Ovarian MDA levels showed a significant increase in the PCOS group compared to those of the control rats (Figure 3A, P < 0.001). Furthermore, in rats receiving 200 and 400 mg/kg *P. anisum*, the levels of MDA diminished compared to those of the PCOS group, with significance observed only in the 400 mg/kg treatment (Figure 3A, P < 0.05).

Compared to controls, the PCOS group had insignificantly lower GPx activity in the ovaries. However, rats treated with 200 and 400 mg/kg doses of *P. anisum* showed a significant increment in ovarian GPx activity compared to the PCOS group (Figure 3B, P < 0.01 and P < 0.001, respectively).

As shown in Figure 3C, ovarian SOD activity was significantly decreased in the PCOS group compared to the control group (Figure 3C, P < 0.05). However, animals receiving 200 and 400 mg/kg *P. anisum* showed significantly increased ovarian SOD activity compared to the PCOS group (Figure 3C, P < 0.001).

### 4.5. Effects of *P. anisum* Extract on Oxidative Stress Biomarkers in Liver Tissue of PCOS-Induced Rats

**Figure 4.**
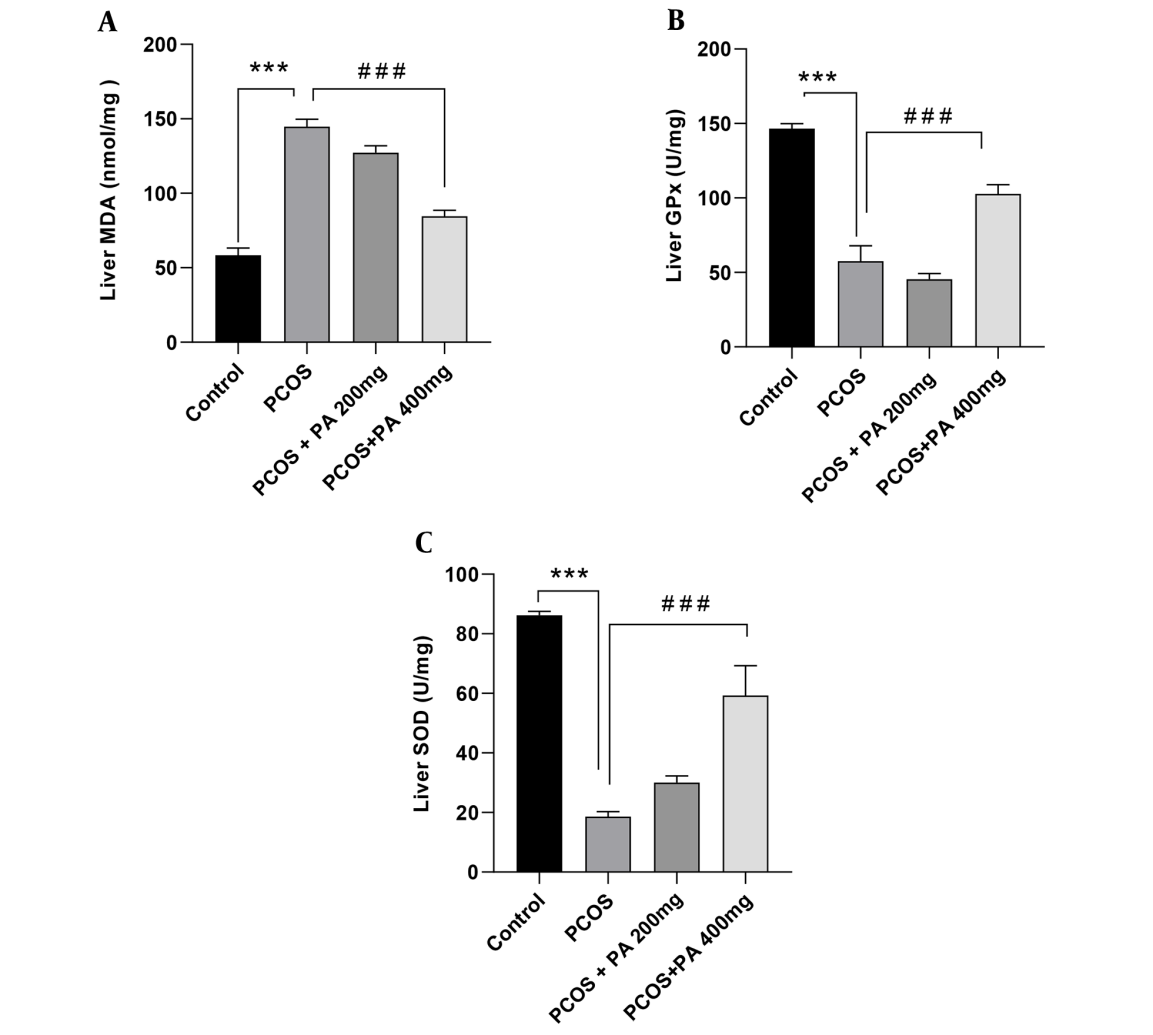
Effect of treatment with *P. anisum* extract (200 and 400 mg/kg, orally, once daily, for 3 weeks) on MDA (A) levels, GPx (B), and SOD (C) activities in liver tissue of PCOS-induced rats. Values are expressed as mean ± SEM (n = 5 per group). Data were analyzed using one-way ANOVA followed by Tukey’s post hoc test. *** P < 0.001 vs. the control group and ### P < 0.001 vs. the PCOS group. PCOS: Polycystic ovary syndrome group; PA: *Pimpinella anisum*.

Following PCOS induction, MDA levels in the liver were significantly higher than controls (Figure 4A, P < 0.001). Administration of *P. anisum* at a dose of 400 mg/kg resulted in reduced levels of MDA compared to the PCOS group (Figure 4A, P < 0.001).

A significant decrease in liver GPx activity was observed in the PCOS group compared to the control group (Figure 4B, P < 0.001). Administration of *P. anisum* at a dose of 400 mg/kg significantly increased GPx activity in the liver compared to the PCOS group (Figure 4B, P < 0.001).

In the PCOS group, SOD activity in the liver was significantly decreased compared to the control group (Figure 4C, P < 0.001). However, *P. anisum* administration resulted in an increment in liver SOD activity compared to the PCOS group, with the dose of 400 mg/kg being more effective than the dose of 200 mg/kg (Figure 4C, P < 0.001).

### 4.6. Effects of *P. anisum* Extract on TNF-α and IL-6 mRNA Expression in Ovarian Tissue of PCOS-Induced Rats

**Figure 5.**
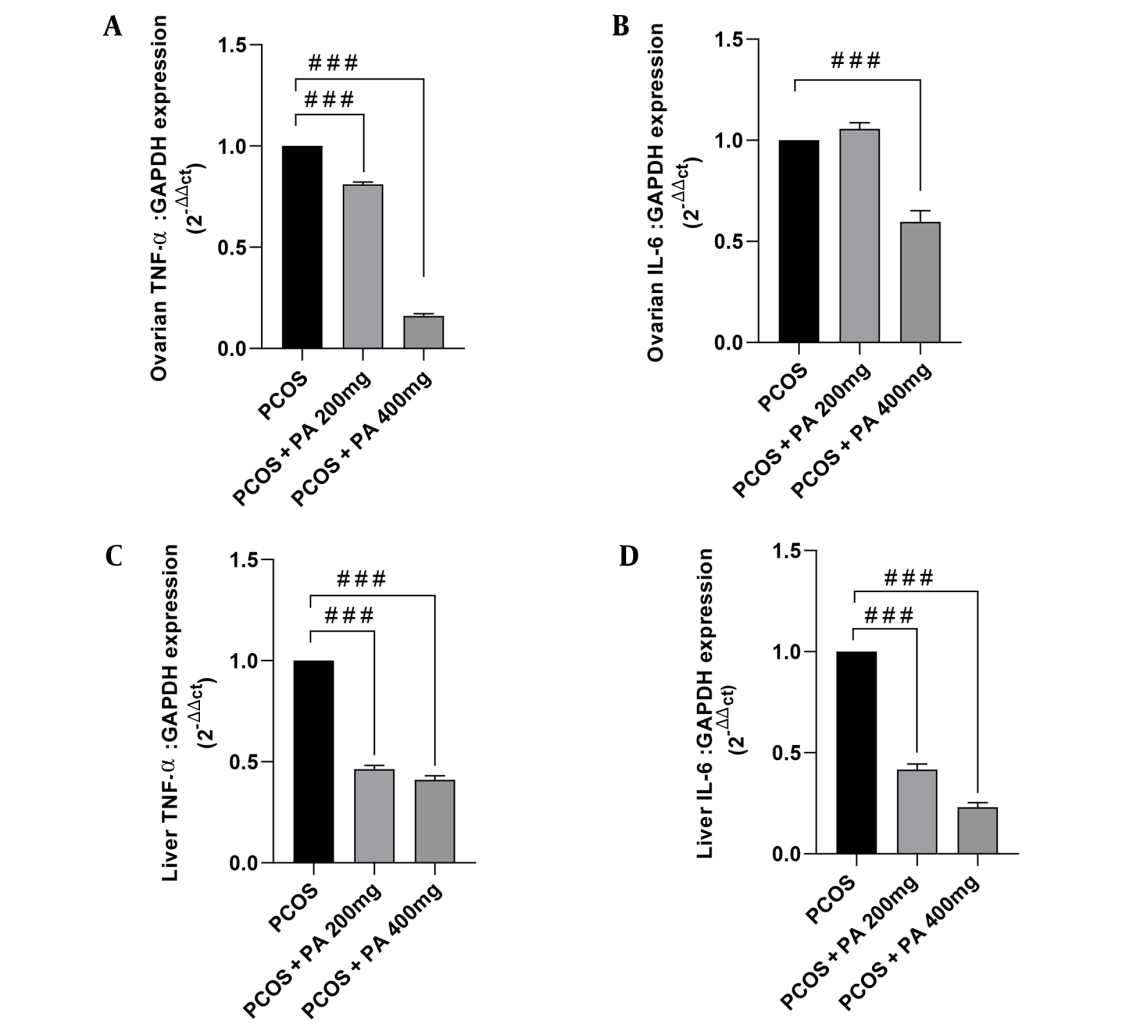
Effect of treatment with *P. anisum* extract (200 and 400 mg/kg, orally, once daily, for 3 weeks) on TNF-α and IL-6 mRNA expression in ovarian (A and B) and liver (C and D) tissues of PCOS-induced rats. Values are expressed as mean ± SEM (n = 3 per group). Data were analyzed using one-way ANOVA followed by Tukey’s post hoc test. ### P < 0.001 vs. the PCOS group. PCOS: Polycystic ovary syndrome group; PA: *Pimpinella anisum*.

Our results showed that ovarian TNF-α mRNA expression levels were drastically reduced in PCOS rats receiving 200 and 400 mg/kg *P. anisum* compared to the PCOS group (Figure 5A, P < 0.001). Moreover, treatment with *P. anisum* exhibited decreased expression of IL-6 cytokine. However, PCOS rats receiving 400 mg/kg *P. anisum* showed a significant difference compared to the PCOS group (Figure 5B, P < 0.001).

### 4.7. Effects of *P. anisum* Extract on TNF-α and IL-6 mRNA Expression in Liver Tissue of PCOS-Induced Rats

Regarding cytokine expression, qRT-PCR analysis revealed that mRNA expression levels of TNF-α and IL-6 in the liver were significantly decreased in treated PCOS rats at the doses of 200 and 400 mg/kg *P. anisum* compared to the PCOS group (Figure 5C and D, P < 0.001).

### 4.8. Effects of *P. anisum* Extract on Folliculogenesis in the PCOS-Induced Rats

**Figure 6.**
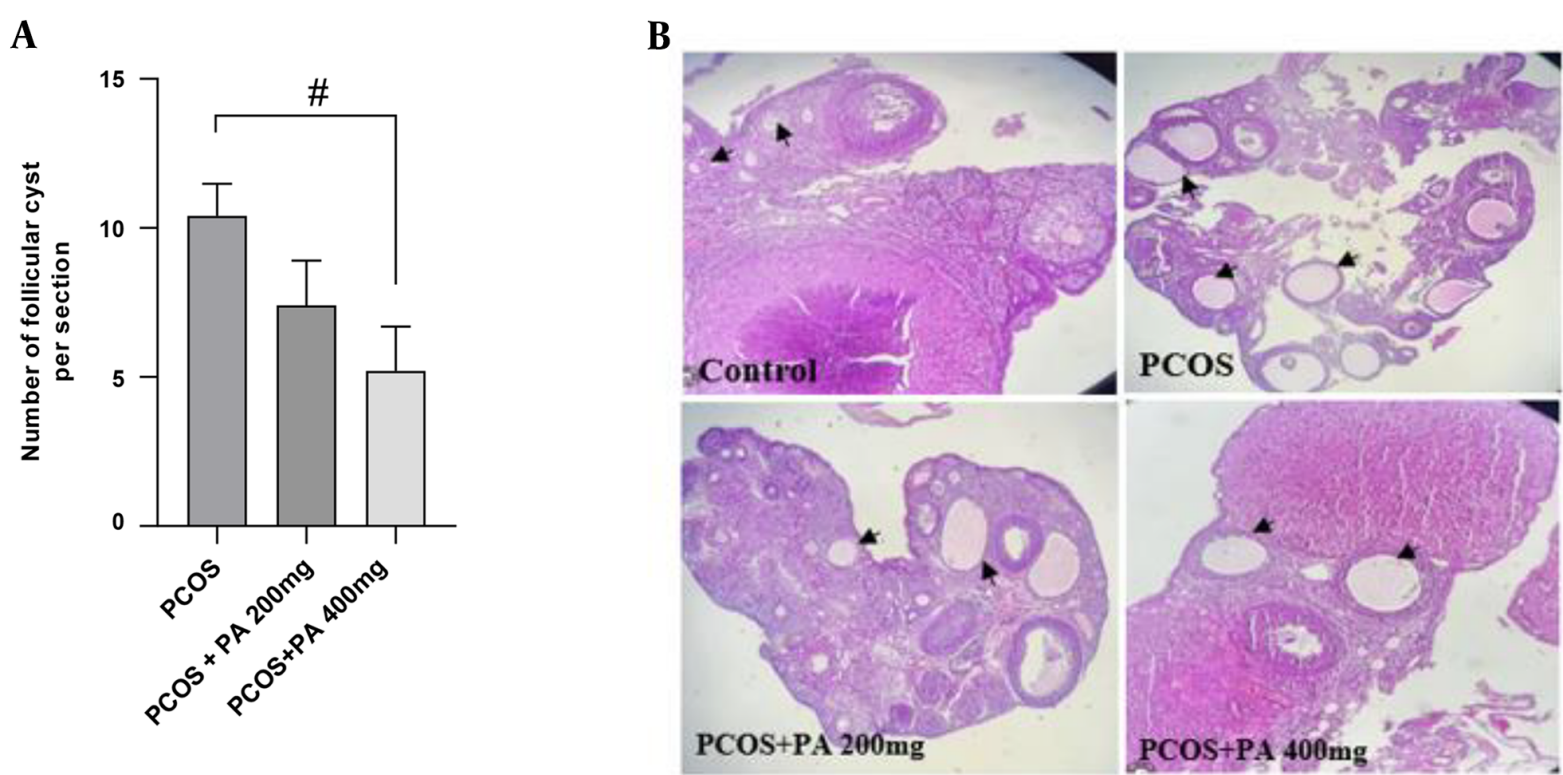
Effect of treatment with *P. anisum* extract (200 and 400 mg/kg, orally, once daily, for 3 weeks) on the number of follicular (A) and ovarian histopathology (B) in the PCOS-induced rats (H&E, × 100). Values are expressed as mean ± SEM (n = 3 per group). Data were analyzed using one-way ANOVA followed by Tukey’s post hoc test. # P < 0.05 vs. the PCOS group. PCOS: Polycystic ovary syndrome group; PA: *Pimpinella anisum*.

The ovaries of the control groups showed normal architecture without any pathological changes. However, histopathological results of the PCOS rats revealed many cystic follicles of different sizes. Interestingly, the number of follicular cysts in the ovaries was significantly reduced in the PCOS + PA 400 mg/kg group compared to the PCOS group (Figure 6A and B, P < 0.05).

## 5. Discussion

The results of this study showed that *P. anisum* could improve the function of the ovarian follicles in PCOS-induced rats in a dose-dependent manner, which was associated with a reduction in oxidative stress markers and pro-inflammatory cytokines including TNF-α and IL-6 mRNA expression levels and their content in both ovary and liver tissues. In addition, *P. anisum* decreased the number of ovarian follicular cysts.

Dysfunction in endocrine metabolism, abnormal androgen hormone levels, and chronic inflammation are observed in PCOS (8). While most studies have focused on the impact of hyperandrogenism on the pathological mechanisms of PCOS, particularly follicular dysplasia and anovulation, inflammation stands out as a critical risk factor among the main contributors to PCOS pathophysiology (35, 36). Several studies have indicated that PCOS is associated with progressive inflammation of the ovarian follicles and a decline in ovarian function (13, 37). IL-6 and TNF-α have been reported as important mediators of the inflammatory response, implicated in the apoptosis or necrosis of ovarian follicles (38). Our experimental data on PCOS also revealed that oxidative stress, characterized by significant ROS formation and antioxidant degradation, plays a critical role in PCOS pathogenesis. The association between oxidative stress status and inflammatory response has been documented in preclinical animal models and women with PCOS (39, 40). Moreover, emerging reports have demonstrated the positive effects of herbal medicines and alternative treatments on PCOS symptoms, particularly in alleviating inflammation and oxidative stress (41, 42). In this context, we assessed the effects of *P. anisum* as a protective agent against PCOS damage on these markers in a rat PCOS model. Many studies have reported that *P. anisum* has great potential as an antioxidant and anti-inflammatory factor due to the presence of phytochemical compounds (e.g., flavonoids and trans-anethole) responsible for the medicinal properties of this plant (43-45).

In the current study, PCOS rats treated with *P. anisum* exhibited lower expression of TNF-α and IL-6 mRNA, along with a decrease in the content of these parameters in ovarian tissue. Additionally, ovarian MDA levels increased while SOD and GPX activity decreased in PCOS rats. To our knowledge, there are no published studies examining the effects of *P. anisum* administration on inflammatory responses and biomarkers of oxidative stress in ovarian tissue of PCOS rats. Consistent with our results, decreased inflammation and restored oxidant/antioxidant status have been reported in other organs after *P. anisum* administration in rat models of various disorders (46-49).

Furthermore, several studies have shown that *P. anisum* and its bioactive compounds can influence gene expression at the cellular level. Dargahi et al. (50) and Iannarelli et al. (51) reported that administration of an aqueous *P. anisum* L. seed extract (0.16 mg/kg/day by oral gavage for 10 days) and aniseed essential oil (incubated with 0.3% of aniseed) reduced mRNA expression of proinflammatory cytokines and suppressed inflammatory responses in mice with ovalbumin-induced asthma and lung cell lines, respectively. Other studies have confirmed the anti-inflammatory activity of trans-anethole (52-54) and flavonoids (55, 56) as main compounds of *P. anisum* by inhibiting gene expression and the release of inflammatory pathways such as IL-6, TNF-α, and nuclear factor kappa beta (NF-κB) under in vitro and in vivo conditions. Additionally, dose-dependent changes in antioxidant activity and lipid peroxidation in PCOS rats may be attributed to the effect of this extract on the oxidative genetic machinery (52).

As discussed, chronic inflammation, oxidative stress, and altered mRNA expression are common features of PCOS (57). Given the close linkage between inflammation and oxidative stress, elevated oxidative stress is usually involved in the development of inflammatory conditions (40). Furthermore, the hepatoprotective effects of natural sources in PCOS have been described in "Effects of natural products on PCOS: From traditional medicine to modern drug discovery" (58). In our study, *P. anisum* exhibited hepatoprotective properties in experimental PCOS model rats, significantly reducing oxidative stress and inflammation damage to the liver, possibly due to its ability to improve liver function (48). The present data showed that *P. anisum* decreased the number of follicular cysts in PCOS rats, which could contribute to altering the gene expression of inflammation markers and reducing the content of inflammatory and oxidative stress markers in ovarian tissue. It is noteworthy that treatment with *P. anisum* at a dose of 400 mg/kg was more effective than at a dose of 200 mg/kg. Together, these findings suggest that extracts with higher concentrations may contain higher levels of bioactive components. In other words, *P. anisum* can be considered an effective therapeutic option for PCOS.

### 5.1. Conclusions

In conclusion, our results showed that dose-dependent administration of *P. anisum* inhibited PCOS-associated production of TNF-α and IL-6, as well as MDA, restoring SOD and GPX activity in PCOS rats. These findings support the use of *P. anisum* as a complementary treatment for suppressing PCOS.
